# High-Content Screening of Eukaryotic Kinase Inhibitors Identify CHK2 Inhibitor Activity Against *Mycobacterium tuberculosis*

**DOI:** 10.3389/fmicb.2020.553962

**Published:** 2020-09-18

**Authors:** Tirosh Shapira, Leah Rankine-Wilson, Joseph D. Chao, Virginia Pichler, Celine Rens, Tom Pfeifer, Yossef Av-Gay

**Affiliations:** ^1^Division of Infectious Diseases, Department of Medicine, The University of British Columbia, Vancouver, BC, Canada; ^2^Department of Microbiology and Immunology, Life Sciences Institute, The University of British Columbia, Vancouver, BC, Canada

**Keywords:** tuberculosis, CHK2, host-directed therapy, kinase inhibitor, screen

## Abstract

A screen of a eukaryotic kinase inhibitor library in an established intracellular infection model identified a set of drug candidates enabling intracellular killing of *Mycobacterium tuberculosis* (*M.tb*). Screen validity was confirmed internally by a *Z*′ = 0.5 and externally by detecting previously reported host-targeting anti-*M.tb* compounds. Inhibitors of the CHK kinase family, specifically checkpoint kinase 2 (CHK2), showed the highest inhibition and lowest toxicity of all kinase families. The screen identified and validated DDUG, a CHK2 inhibitor, as a novel bactericidal anti-*M.tb* compound. CHK2 inhibition by RNAi phenocopied the intracellular inhibitory effect of DDUG. DDUG was active intracellularly against *M.tb*, but not other mycobacteria. DDUG also had extracellular activity against 4 of 12 bacteria tested, including *M.tb*. Combined, these observations suggest DDUG acts in tandem against both host and pathogen. Importantly, DDUG’s validation highlights the screening and analysis methodology developed for this screen, which identified novel host-directed anti-*M.tb* compounds.

## Introduction

Tuberculosis (TB) caused by *Mycobacterium tuberculosis* (*M.tb*) infects one quarter of the world’s population and kills 1.5–2 million people annually, with a global case fatality rate of 16% and a poor treatment success rate of 55% for multidrug resistant TB (WHO, 2018). Current treatment for tuberculosis involves a cocktail of four first-line drugs including rifampicin, isoniazid, ethambutol, and pyrazinamide for 6–9 months (CDC, 2016), often involving patient isolation in treatment facilities specializing in tuberculosis management. To further exacerbate the threat TB places on global health and economy, global rates of multidrug resistant *M.tb* cases has been rising considerably (WHO, 2018). Second-line drugs are intended to be used sparingly due to decreased efficacy and greater toxicity-associated complications, as well as to limit the emergence of extensively drug resistant strains. The development of safer drugs and the use of synergistic drugs to reduce drug concentrations and subsequent toxicity has been in the forefront of the global campaign against TB. Identifying the mechanisms of action of current drugs, as well as understanding the mechanisms of host-pathogen interactions between *M.tb* and alveolar macrophage to generate new targets for drug intervention, is fundamental to this effort (Parish, 2020).

*Mycobacterium tuberculosis*’ pathogenicity is strongly associated with its long co-evolution with its obligate human host, which allowed the development of mechanisms of subverting host defenses to permit its successful infection, persistence, and dissemination (Poirier and Av-Gay, 2012; Hmama et al., 2015). *M.tb* primarily infects, survives, and replicates inside human macrophages, and it evades host phagocytic activity via a series of kinase and phosphatase effectors, among other methods (Poirier and Av-Gay, 2012). The most notable of these is the secreted protein tyrosine phosphatase PtpA, which inhibits both phagosome acidification and phagosome-lysosome fusion (Bach et al., 2008; Wong et al., 2011). By tracing the activity of PtpA, the macrophage’s GSK3 was discovered as a prominent host target of PtpA (Poirier et al., 2014). Anti-inflammatory agents such as the non-steroidal anti-inflammatory drugs (Kroesen et al., 2017) and signaling modulation agents such as metformin (Padmapriyadarsini et al., 2019) provide a promising avenue for a new line of adjunctive TB therapeutics termed host-directed therapies (HDTs). HDTs are exciting for their potential to treat drug-resistant *M.tb*, synergistically enhance the activity of current *M.tb* drugs, and may not be susceptible themselves to resistance development (Tobin, 2015).

Current anti-tuberculosis treatments have been developed against extracellular bacteria without the consideration for intracellular infection dynamics or host responses outside general toxicity testing. For example, the most recently FDA-approved TB compound, pretomanid, developed in 1993 (Ashtekar et al., 1993), was initially tested *in vitro* and *in vivo* only, with *ex vivo* (via macrophage infection) analytics being overshadowed. New developments of intracellular screening technologies have made *ex vivo* screening a powerful tool for high throughput drug discovery, taking into consideration the first and arguably most important infection niche (Sorrentino et al., 2016; Parish, 2020). Another historic hurdle to overcome in development of drugs against *M.tb* is the use of non-*M.tb* models for top-down screening due to their simpler upkeep requirements. However, drugs have proven to have significantly different activities against different mycobacteria family members, as in the case of pretomanid, which shows no activity against the closely related *Mycobacterium avium* and *Mycobacterium intracellulare* (Ashtekar et al., 1991). In compound screening campaigns, compromising on screening models that are remote from the pathogen (*M.tb*) and its host (human macrophages) can generate false negatives, i.e., hit compounds that fail “hit-to-lead” development, and false positives, i.e., rejection of compounds that may be active if the screening would have presented a biologically relevant model. One major hurdle in screening campaigns for new therapeutics against *M.tb* is the expensive and labor-intensive requirements of Biological Contamination Level (BCL) 3 facilities. The BCL2-safe MC^2^ 6206 auxotrophic strain of *M.tb* (ΔleuDΔpanCD), originally developed as a vaccine model (Sampson et al., 2004), was previously used successfully to test anti-*M.tb* compounds (Schaaf et al., 2016; Mouton et al., 2019).

With the pressing need for new drugs to combat the TB pandemic and development of technologies that allow accurate testing of intracellular *M.tb* infection models, HDTs have been increasingly investigated in recent years (Kolloli and Subbian, 2017). A study that investigated RNAi targeting host kinases and their effect on intracellular *M.tb* growth in mouse macrophages uncovered a variety of important host pathways crucial for infection (Jayaswal et al., 2010) including the TGFβR1 and CDC25A signaling networks, and this was corroborated in a human macrophage screen (Kumar et al., 2010). Screening of prospective HDTs has been accelerated by the development of anti-cancer therapeutics libraries, with a multitude of libraries available for academic and commercial use (Babichev et al., 2016). Additionally, high-content screening (HCS) allows for reliable and valid data acquisition from macrophages infected vs. uninfected with *M.tb* (Sahile et al., 2020). Additionally, the development of the THP-1 human macrophage model as a validated model for human primary macrophages has allowed for high throughput advances (Johnson and Abramovitch, 2015; Madhvi et al., 2019). This study aimed to harness all these advancements to identify novel anti-*M.tb* HDTs and characterize the networks of host kinases important for inhibition of intracellular *M.tb*.

## Materials and Methods

### Bacterial and Mammalian Strains and Culturing

*Mycobacterium tuberculosis* strain H37Rv, *Mycobacterium abscessus* ATCC 19977T, R (rough form), and *Mycobacterium bovis*-BCG were all transformed with the pTEC27 fluorescent reporter plasmid, harboring a tomatoRFP and hygromycin resistance gene (Bernut et al., 2014; Cambier et al., 2014). All the mycobacterial strains were routinely grown in 7H9 broth (Difco Middlebrook) supplemented with 10% (v/v) OADC (5% bovine albumin fraction, 2% dextrose, 0.004% catalase, 0.05% oleic acid, and 0.8% sodium chloride solution), 0.05% (v/v) Tween-80 (Sigma-Aldrich), and 50 μg/mL hygromycin B at 37°C in standing cultures. Other bacteria used are listed in Table 3 with their strain number and were cultivated according to ATCC recommendations.

The THP-1 cells (ATCC TIB-202^TM^) used are derived from human monocytes obtained from a patient with acute monocytic leukemia. THP-1 cells were grown in complete RPMI1640 medium (5% FBS, 2% glutamine, 1% non-essential amino acids, and 1% penicillin and streptomycin). Cells were grown in T75 flask with 5% carbon dioxide (CO2) at 37°C. Cell density was kept between 0.25 and 1 × 10^6^ cells/mL. Cultures were used for up to three months. For assays, RPMI1640 medium with all supplements but antibiotics (incomplete RPMI1640 medium) was used. Bone marrow derived macrophages (BMDM) were kindly provided by P. Johnson and M. Dosanjh from C57BL/6, produced using 500 UI/mL M-CSF in DMEM.

### Compounds

A duplicated OICR Kinase library at 10 μM was the kind contribution of the Centre for Drug Research and Development (now adMare, BC, Canada). Imatinib (G40700), 10-DEBC (D298368), and Tandutinib (T006550) were purchased from TRC Canada (ON, Canada). DDUG (SML0781) was purchased from Sigma-Aldrich (MO, United States). Bedaquiline (TMC-207) was purchased from AddoQ (CA, United States). All compounds were solubilized in DMSO at a concentration of 10 mM.

### Infection

Bacterial culture grown to log phase was centrifuged (4000 rpm, RT for 10 min) and washed once in 7H9 media containing 0.05% Tween 80 (20%). The supernatant was discarded after centrifugation and the pellets were then re-suspended in RPMI1640 medium, de-clumped using a 25G blunt syringe, and OD_600_ was measured (OD_600_ of 1 ≈ 3.3 × 10^8^ CFU/mL). Immediately before the infection the bacterial suspension was opsonized by adding non-decomplemented 10% human serum and incubating for 30 min at 37°C. A cell suspension of THP-1 cells (1 × 10^6^ cells/mL) in incomplete RPMI1640 was incubated with the opsonized *M.tb* single cells suspension at a Multiplicity of Infection of 2:1, and phorbol12-myristate13-acetate PMA (40 ng/mL) for 4 h at 37°C under constant agitation, as previously described (Sorrentino et al., 2016). After infection, the THP-1 cell suspension was centrifuged (750 rpm, RT, 10 min) and washed with RPMI1640 3 times. After the final wash, cell suspension was dispensed onto 96-well plates (clear, flat bottom), at a concentration of ∼50,000 THP-1 cells/well. Infected cells plus compounds were incubated for 4 days at a volume of 100 μL/well at 5% CO_2_ and 37°C. After incubation, cells were fixed with paraformaldehyde (PFA, 4% in warm PBS buffer) for 60 min (*M.tb*) or 30 min (*M. abscessus*, *M. bovis*-BCG), fixative removed, and stained with 1 μg/mL Hoechst 33342 in RPMI1640 for 10 min. Hoechst stain was removed, and cells kept in 100 μL RPMI1640. Plates were kept covered in aluminum foil until scanning to avoid photobleaching.

Bone marrow derived macrophages (BMDM) were identically used, with the sole difference of using DMEM medium rather than RPMI1640 medium.

*Salmonella typhimurium* transformed with a luminescent reporter plasmid, pCS26-Pac (Bjarnason et al., 2003), was grown overnight on a Luria-Bertani (LB) agar plate. A broth culture was started several hours prior to the infection. Once the culture reached an OD_600_ of 1, bacteria were pelleted by centrifugation and washed three times with RPMI media. The bacteria were then opsonized for 30 min at 37°C with 10% human serum. The opsonized bacteria were diluted in RPMI up to 1 × 10^6^ CFU/mL and further incubated with THP-1 macrophages seeded at 1 × 10^5^ cells per well in a 96-well plate, differentiated with PMA (40 ng/mL) for a 24 h prior to the infection. After 30 min of incubation, the infected cells were washed three times with fresh RPMI and incubated for an additional hour with 100 μg per mL of gentamicin to kill remaining extracellular bacteria. The infected cells were incubated with the tested compounds in presence of 10 μg/mL of gentamicin. The intracellular growth of the bacteria was assessed after 72 h of infection using luminescence. In order to normalize the bacterial growth to the number of surviving macrophages after 72 h of infection, THP-1 cells were counted using an HCS Platform as detailed below.

### High Content Screening Methodology and Parameters

Monitoring of the intracellular bacterial growth and eukaryote nuclei was performed using the CellInsight CX5 High Content platform. First, the THP-1 nuclei are identified and counted using the 350/461 nm wavelength (Hoechst 33342); cell debris and other particles are deducted based on a size filter tool. Following that a Region of Interest (ROI, or “circle”) is drawn around each host cell nuclei and validated against the brightfield image to correspond with most cell membranes. The ROI encompass where 533/588 nm wavelength (pTEC27, or “spots”) are located. Finally, the software identifies, counts, and measures the pixel intensity of the “spots” within the “circle.” The fluorescent spot intensity measured within each cell (circle) is then added and quantified for each well using Thermo Fisher Scientific^TM^ HCS Studio^TM^ Cell Analysis Software. The total circle spot intensity of each well corresponds to the intracellular bacterial load. We (Richter et al., 2019) and others have previously validated these fluorescent measurements closely correspond with CFUs. Nuclei stain (Hoechst 33342) was used to quantify THP-1 cell loss (due to cytotoxicity or loss of adherence).

### Screening

A compound screen strategy utilized the Ontario Institute for Cancer Research Kinase Inhibitor library (Babichev et al., 2016) was kindly supplied by the Centre for Drug Research & Development, BC, Canada. Library was screened in duplicate, at a single dose-concentration of 10 μM. Before the screen, a Z′ calibration was performed testing rifampicin and bedaquiline (data not shown), after which bedaquiline at a concentration of 4 μM was chosen as the positive control, while the compound solvent, DMSO was used as a negative control at a concentration of 1%. Z′ were tested for each individual compound plate, with an average Z′ of 0.5 (±0.1) using the “CircSpotTotalInten” readout. The Z′ factor was determined using the formula:

$$Z^{\prime }=1-\frac{3\,\left({\text{SD}}_{\text{bedaquilin}}+{\text{SD}}_{\text{DMSO}}\right)}{\left({\text{M}}_{\text{bedaquilin}}-{\text{M}}_{\text{DMSO}}\right)}$$

where SD is the standard deviation and M is mean.

For compound targets, close family-members were clustered together. For example, checkpoint kinase 1 (CHK1) and CHK2 were clustered under the CHK family, and VEGFR1, 2, and 3 were clustered under the VEGFR family. Target family average activities, average toxicities, filtering, and graphing were preformed using JMP^®^, version 14 (SAS Institute Inc.).

### Intracellular and Broth Dose-Response, Disk Diffusion Test

Intracellular dose-response of bacterial load (pTEC27 intracellular signal) was performed at dilution factors of 1:1, at range 0.2 μM < (C) < 50 μM, with at least three biological replications per concentration (4 ≤ *n* ≤ 8 technical replication). Bacterial loads were interpolated to negative control (1% DMSO) = 0, and positive control (4 μM bedaquiline) = 100. GraphPad Prism 6 (GraphPad Software, Inc.) non-linear regression fit modeling variable slope was used to generate a dose-response curve [Y=Bottom + (Top−Bottom)/1 + 10^(*LogIC50*−X)×HillSlope^], constrained to top = 100, bottom = 0.

Broth dose-response performed at similar concentrations and replications, using 0.02% resazurin sodium salt (B21187.06, Alfa Aesar Chemicals, MA, United States). Bacteria strains listed in Table 3 and cultivated using strain-appropriate culturing methods. To apply identical controls for all bacteria tested, a cocktail of 10 μM apramycin, vancomycin, and gentamicin was used as a positive control, and water as negative control.

Disk diffusion assay based on the Kirby-Bauer method (Bauer et al., 1966) using 10 μg DDUG, disks of 10 μg apramycin, vancomycin, or gentamicin as positive controls, and water disk as negative control. Sensitivity (%) calculated based on gentamicin, as the active control in all strains who showed a DDUG effect. The experiment was performed in triplicate.

### RNAi Interference

THP-1 cells were seeded at 50,000 cells per well in 96-well clear flat bottom plates and differentiated overnight in incomplete RPMI1640 media with 40 ng/mL PMA to ensure RNAi treatment is performed on fully differentiated cells. Cells were transfected using 10 pmole siRNA (IDT) and 2 μL of HiPerFect transfection reagent (Qiagen, Germany) per well, following the supplied protocol. Cells were infected with *M.tb* after 24 h. RNAi inhibition was not verified with qPCR due to COVID-19 efforts, resulting in lack of reagents.

## Results

### HCS Reveals Established and Novel Active Compounds Against Intracellular *M.tb*

To identify host-targeting anti-*M.tb* compounds, we screened the OICR Kinase library, a small library of kinase inhibitors at various stages of clinical trials, or those used as molecular tools in kinase-inhibitory studies. Our strategy used an established THP-1 human macrophage model (Madhvi et al., 2019), infected with fluorescence-expressing *M.tb*, automatically analyzed and quantified using HCS (Supplementary Figure 1). Screening of the OICR Kinase library revealed that 32 out of 400 compounds showed inhibition of *M.tb* intracellular growth >50% compared to that of 4 μM bedaquiline positive control (Table 1 and Supplementary Table 1).

**TABLE 1:** Criteria used to filter screen hits.

	**Yes (%)**	**No (%)**
Inhibition >50%	32 (8)	368 (92)
Cell loss <30%	169 (42)	231 (58)
Negative *z*-score	56 (14)	344 (86)
Duplicate	365 (91)	35 (9)

**FIGURE 1:**
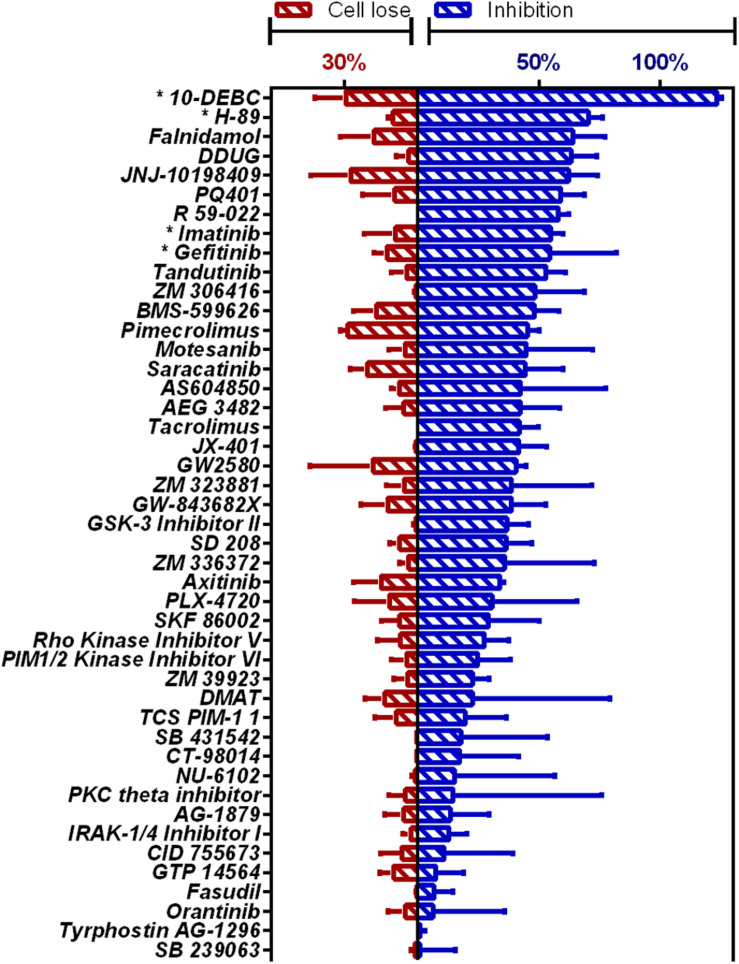
HCS of OICR Kinase Inhibitor library against intracellular *M.tb*. Inhibition of *M.tb* growth (blue), interpolated to the negative control (1% DMSO) set at 0% inhibition and the positive control (4 μM bedaquiline) defined as 100% inhibition. Cell loss (red), normalized to negative control. Previously identified host-targeting *M.tb* inhibitors marked with asterisk (*). Means from compounds with negative *z*-score and cell loss <30% (*n* = 45) are graphed. Error bars represent the SD from two independent screens. Full list of compounds and *z*-scores are tabled in Supplementary Table 2.

Novel compounds highly active against *M.tb* in our assay (Figure 1 and Supplementary Table 2) were Falnidamol, targeting host epidermal growth factor receptor (EGFR); DDUG, targeting host CHK2; JNJ-10198409, targeting host platelet-derived growth factor receptor (PDGF-R); PQ401, targeting host insulin-like growth factor 1 receptor (IGF-1R); R 59-022, targeting diacylglycerol kinase (DAG); and Tandutinib, targeting host fms-like tyrosine kinase 3 (FLT3), KIT, and platelet-derived growth-factor receptor (PDGFR).

Additionally, our assay identified many compounds previously shown to inhibit *M.tb* growth in various other intracellular models of infection. For example, 10-DEBC inhibits *M.tb* in macrophages differentiated from human embryonic stem cells (Han et al., 2019); H-89 inhibits *M.tb* in primary human macrophages (Kuijl et al., 2007); Imatinib in J774A.1 murine macrophage-like cell line (Napier et al., 2011); and Gefitinib also in J774 (Stanley et al., 2014; Sogi et al., 2017).

Our HCS assay enabled us to simultaneously monitor the effect of candidate drugs on bacterial growth and the host cells’ viability, measured as intracellular fluorescence and loss of host cells, respectively (Supplementary Figure 2). As such, although the effect of compounds that eradicate host cells also reduce bacterial load, we were able to distinguish between the two.

### Kinase Target Families Show Different Inhibition of Intracellular *M.tb*, With Clear Link Between Toxicity and Activity

**FIGURE 2:**
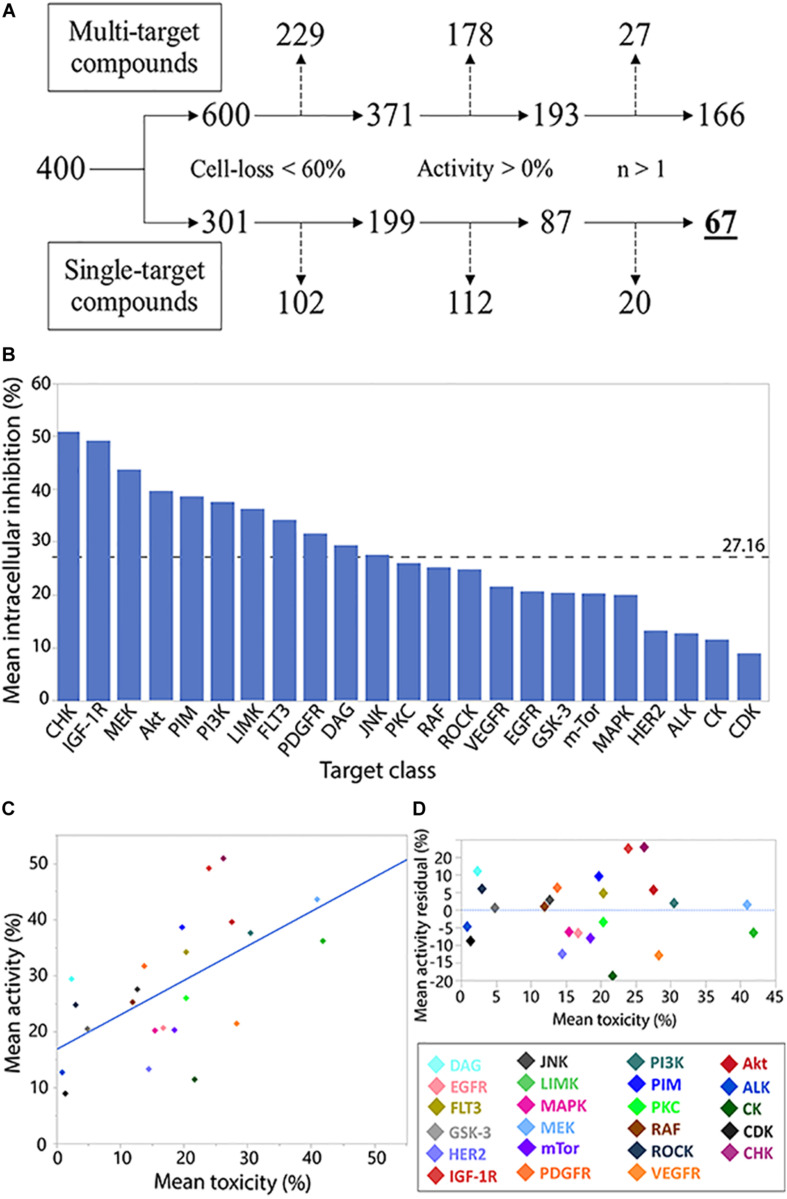
Analysis of compound target class/family. Flowchart analysis of the logic steps taken to ensure activity characterization (measuring normalized intracellular bacterial growth inhibition) of target families was gathered from non-pleiotropic compounds (single-target compounds) and were not skewed by THP-1 cell-loss, exhibit anti-*M.tb* activity, and were represented by more than 1 compound in the library. Branch shifts from compound numbers (400) to compound target numbers (600/301). Excluded targets from each step marked by dashed arrows **(A)**. Compound family charted by mean inhibitory activity. Weighted average marked by dashed line **(B)**. Regression analysis of family toxicity (cell-loss) on activity resulted in linear regression with RMSE = 9.66% (*R*^2^ = 0.36) **(C)**. Analysis of residual activity by target family classes. Positive or negative residual values represent higher or lower activity, respectively, than can be explained by toxicity effects against THP-1 cells **(D)**.

While allowing identification of specific inhibitory compounds and their host toxicity, using a library with well-defined compounds allows investigation of the compound families as well. Following similar, biologically relevant, filtering parameters for individual compound analysis (Figure 2A), we investigated the intracellular *M.tb* growth inhibition (activity) and host cell loss (toxicity).

In the activity and toxicity analysis, we chose library compounds that were characterized as inhibiting only single targets. The average activity across represented kinase families was 27.16% (Figure 2B), with the CHK family of inhibitors representing the highest mean activity.

Many of the library compounds were developed to exert toxicity to cancerous cells and are hypothesized to have a similar effect on the leukemia-derived THP-1 cell line. As our assay measures intracellular bacteria, such measurements can be biased by host-cell toxicity. We therefore set to test the relation between target activity and its toxicity (Figure 2C). A regression fit of *R*^2^ = 0.36 and RMSE of 9.66% suggests for many compounds’ targets, increase in toxicity was correlated with increase in activity and can partially or completely explain the observed activity.

Compound targets residual analysis (Figure 2D) exposed targeting the CHK, IGF-1R, and DAG kinases resulted in activity higher than can be expected from toxicity, while targeting casein kinases (CK), PDGF-R, and human epidermal growth factor receptor 2 (HER2) displayed much lower activity relative to the levels of associated toxicity. Of note, from the top 10 active compounds, Tandutinib is the only novel compound targeting multiple kinases, including FLT3 (+5% residual) and PDGF-R (−12% residual), while the rest are single-target inhibitors.

### Confirmation of the CHK2 Inhibitor DDUG as a Host-Targeting *M.tb* Inhibitor

**FIGURE 3:**
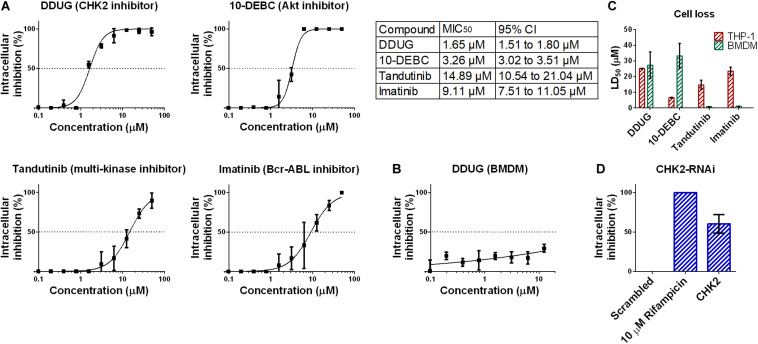
Confirmation of DDUG inhibition of *M.tb* growth in infected THP-1 cells. Dose-dependent inhibition of *M.tb*:THP-1 intracellular growth by novel and established compounds, normalized to 1% DMSO negative control (0% inhibition) **(A)**. CI, confidence interval. DDUG inhibition of *M.tb* growth in infected bone marrow derived macrophages (BMDM) cells **(B)**. Dose-dependant cell loss of THP-1 (red) and bone marrow derived macrophages (BMDM) cells (green) **(C)**. *M.tb* intracellular growth inhibition by RNAi suppression of the DDUG target, CHK2, interpolated to the negative control (1% DMSO) set at 0% inhibition and the positive control (4 μM bedaquiline) defined as 100% inhibition **(D)**. Each data point is an average of 4 independent trials, and error bars are the SEM.

To confirm the HCS results, we chose to focus on DDUG, a CHK2 inhibitor displaying low host cell loss but high level of inhibition of *M.tb* intracellular growth. We also examined Tandutinib as multi-target inhibitor, and Imatinib and 10-DEBC as controls. Dose-dependant analysis of the effect of the selected compounds against intracellular *M.tb* (Figure 3A) confirmed the HCS results and demonstrated, as previously reported, that host-targeting compounds can also exert their inhibitory effect in a dose-dependant manner like antibiotics (Stanley et al., 2014). DDUG’s MIC_50_ values were approximately twice as inhibitory as those of 10-DEBC, indicating the high inhibition by 10-DEBC in the screen might be somewhat biased by high cell loss. DDUG was not active against *M.tb* in mouse primary macrophages (Figure 3B).

To validate cell-loss HCS results, we examined cell loss in THP-1 and in mouse bone-marrow derived macrophages (BMDM) (Figure 3C). Like inhibition, HCS results were confirmed in dose-dependant analysis. In BMDM, DDUG and 10-DEBC showed low cell loss, corresponding to ∼16- and 10-fold higher, than their respective MIC_50_ values. While DDUG cell loss was comparable between primary and immortalized cells (LD_50_ = 25.1 μM and LD_50_ = 27.2 μM, respectively), 10-DEBC’s stark cell loss in THP-1 cells was almost completely nullified (LD_50_ = 6.5 μM and LD_50_ = 33.3 μM, respectively). As 10-DEBC showed no significant toxicity in macrophages differentiated from primary stem cells (Han et al., 2019), it is likely that 10-DEBC is specifically toxic to the THP-1 model.

DDUG is a selective inhibitor of the cell-cycle regulator kinase CHK2. CHK2 suppression using RNAi was able to inhibit intracellular *M.tb* growth with inhibition levels equivalent to those found in the screen (∼10 μM, Figure 3D), suggesting host CHK2 inhibition may explain the inhibitory potential of DDUG and providing validation to the small compound approach using a gene-knockdown approach.

### DDUG Is Bactericidal and Specific to Intracellular *M.tb*, While 10-DEBC Is Bacteriostatic and Inhibits Other Intracellular Mycobacteria Species

**FIGURE 4:**
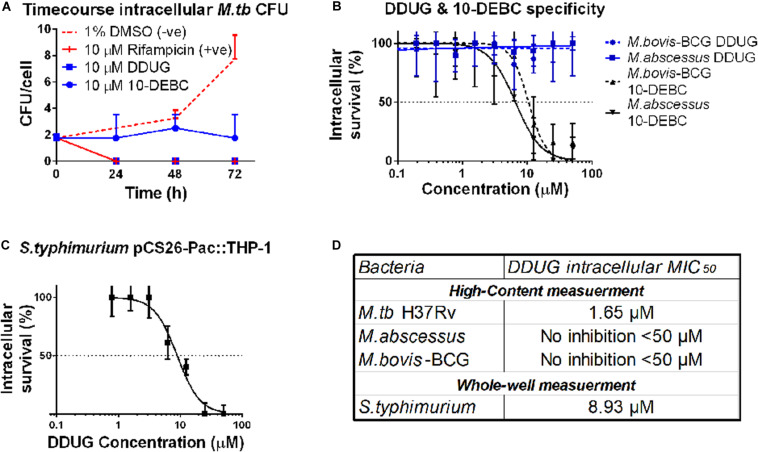
DDUG Mode of intracellular inhibition and Mycobacteria specificity. Intracellular CFU calculated by normalizing to number of THP-1 cells, treated with DDUG (blue squares) or 10-DEBC (blue circles) or controls (red) **(A)**. Compound specificity was compared by challenging pathogenic mycobacteria (*M. abscessus*, solid line; *M. bovis*-BCG, dotted line) with DDUG (blue) or 10-DEBC (black) in an intracellular dose-dependency assay, normalized to 1% DMSO (0% inhibition) and 4 **μ** M bedaquiline (100% inhibition) **(B)**. DDUG inhibition of luciferase-expressing *S. typhimurium* growth in infected THP-1 cells **(C)**. Error bars represent the SEM from four **(A,B)** or six **(C)** independent trials. Comparison of DDUG intracellular effect on different bacteria **(D)**.

To test whether DDUG is bacteriostatic or bacteriolytic to *M.tb* during intracellular infection, a time-course colony-forming units (CFU) assay of *M.tb* in the presence of 10 μM DDUG was performed, and 10-DEBC was similarly tested, as this fundamental information was not reported. DDUG showed bactericidal properties similar to that of the positive rifampicin bactericidal control, while 10-DEBC seemed to act as a bacteriostatic compound with no significant change in CFU/cell in a span of 4 days, where the untreated control replicated twice (Figure 4A).

To test if these compounds are specific to *M.tb* or might be affecting mycobacteria in general, we infected THP-1 with the fast-growing *M. abscessus* and the vaccine strain, *M. bovis*-BCG, harboring the same pTEC27 fluorescent reporter plasmid. DDUG’s intracellular activity specifically inhibited *M.tb* and had no inhibitory effect on the growth of the other mycobacteria tested (Figure 4B) compared to the untreated control. Conversely, 10-DEBC was found to be a general inhibitor against mycobacteria, inhibiting both *M. abscessus* and *M. bovis*-BCG, each with a MIC_50_ of approximately 10 μM (Figure 4B). DDUG was also able to inhibit another intracellular bacteria, luciferase-expressing *S. typhimurium*, at a MIC_50_ = 8.9 μM (Figure 4C), ∼5-fold less intracellular inhibition than with *M.tb* (Figure 4D).

### DDUG and Other Host-Directed Therapies Are Effective Against the *M.tb* Auxotroph

To examine if the BCL2-safe MC^2^ 6206 auxotrophic strain of *M.tb* may be a plausible model for host-targeted compound screens, we tested if the auxotroph is sensitive to DDUG and the previously characterized HDTs Imatinib and 10-DEBC. The auxotroph was indeed inhibited intracellularly by all three compounds, with MIC_50_ absolute difference values relative to those in *M.tb*, ranging between 0.4 and 6.71 μM (Table 2).

**TABLE 2:** Activity of host-directed compounds on intracellular *M.tb* strain MC^2^ 6206.

**Compound**	**MIC_50_**	**95% Confidence interval**	**H37Rv MIC_50_**	**Absolute difference**
DDUG	3.8 μM	3.4–4.4 μM	1.65 μM	2.15 μM
10-DEBC	2.9 μM	2.3–4.2 μM	3.26 μM	0.36 μM
Imatinib	2.4 μM	2.1–2.8 μM	9.11 μM	6.71 μM

*Dose-response regression generated from three independent trials.*

### DDUG Antibacterial Activity *in vitro*

Some host-targeting compounds have been demonstrated to possess activity against bacterial kinases, such as the cyclin-dependent kinase 1 inhibitor NU-6027 (Kidwai et al., 2019) and the CHK1 inhibitor AZD7762 (Kanehiro et al., 2018), which inhibit the *M.tb* kinase, PknG. To examine if DDUG can inhibit bacterial growth in broth, disk diffusion and resazurin assays were conducted, examining a range of pathogenic bacteria and *M.tb* (Table 3). DDUG inhibited *M.tb* growth at concentrations similar to those displayed intracellularly (Tables 2, 3). In addition, DDUG showed inhibitory activity against three of the 12 tested bacteria, including both Gram-positive (*Bacillus subtilis* and *Staphylococcus epidermidis*) and Gram-negative (*Moraxella catarrhalis*) members, without obvious unifying characteristics. *M. catarrhalis* was the most sensitive to DDUG with an MIC50 of less than 1.6 μM. Overall, 75% of the tested bacteria were not inhibited by DDUG in broth and 80% were not inhibited on solid media, including *M. abscessus*. Despite the reproducible activity of DDUG against *M.tb* in broth, DDUG activity in agar was inconsistent, with repeated failures to generate a DDUG-resistant mutant on plates for bacterial target identification.

**TABLE 3:** DDUG broth activity against a range of pathogenic bacteria.

**Bacteria**	**Disk diffusion**	**Resazurin (MIC)**
*Acinetobacter baumannii* BAA-747	R	R (MIC > 50 μM)
*B. subtilis* ATCC 6633	R	S (MIC = 9.4 μM ± 2.7 μM)
*Escherichia coli* ATCC 25922	R	R (MIC > 50 μM)
*Enterococcus faecalis* ATCC 29212	R	R (MIC > 50 μM)
*M. abscessus* ATCC 19977T (R)	R	R (MIC > 50 μM)
*M. catarrhalis* ATCC 25240	S (19% ± 2%)	S (MIC < 1.6 μM)
*M. tuberculosis* H37Rv	N/A	S (MIC 3.125 μM)
*Pseudomonas aeruginosa* ATCC 14210	R	R (MIC > 50 μM)
*Staphylococcus aureus* ATCC 25923	R	R (MIC = 50 μM)
MRSA ATCC 700698	R	R (MIC = 50 μM)
*S. epidermidis* ATCC 35984	S (7% ± 2%)	S (MIC = 6.25 μM)
*Salmonella typhi* ATCC 13311	R	R (MIC > 50 μM)

*R, resistant; S, sensitive; MIC, minimal inhibitory concentration; N/A, not available. Disk sensitive percent measures the diameter of the zone of inhibition relative to the positive control.*

In summary, by screening a kinase inhibitor library with well-characterized targets using a validated and scalable THP-1:*M.tb*-pTEC27 screen, we were able to reproduce findings from previous reports in other infection models, as well as identify both specific targets and target families that highly inhibit *M.tb* intracellular growth. We confirmed and validated one of the novel compounds, DDUG, and characterized its bactericidal activity, its specificity to *M.tb*, and the auxotrophic BCL2-safe MC^2^ 6206 strain, and we tested its activity in broth against an array of pathogenic bacteria.

## Discussion

In this campaign we demonstrated the power of utilizing a combination of validated and reliable tools, namely THP-1 cells, fluorescent *M.tb*, HCS, and a well-characterized library, to identify and validate potential HDTs. Overall ∼3% of screened compounds answered “yes” to all our hit criteria (Table 1), showcasing that targeting host kinase signaling is a valid approach for novel HDT discovery in TB. A similar screen conducted on an embryonic cell derived macrophage (iMACs) model also reported a ∼3% hit rate (Han et al., 2019). While similar in outcome, THP-1 screening in comparison to iMACs is well established, highly reproducible, and much simpler and cheaper to carry out. Previous screening campaigns in non-human macrophage models, such as carried out *in vitro* (Pethe et al., 2013) and in infected mouse macrophages (Stanley et al., 2014), both reported a ∼0.08% hit rate. Our ability to identify many previously reported anti-*M.tb* host targeting compounds identified in different screening campaigns and the relatively high hit rate together support the use of THP-1 cells for kinase inhibitor screens.

One limitation of this host-cell model of infection is that THP-1 cells are derived from cancerous leukemia. Kinase inhibitors were developed primarily to target cancerous cells, and may therefore be markedly toxic to THP-1 cells. By simultaneously measuring inhibitory activity and cell loss, which can be caused by compound toxicity or compound modulation of cellular adherence, we are able to partially predict compound toxicity and, more importantly, quality control the main outcome measure to avoid bias by host cell count. The main benefit of screening compounds at various stages of clinical trials, as well as tool compounds, is their potential for quick development compared to unknown compounds, with the added benefit that toxicological data exists for most of these compounds.

A secondary analysis of kinase inhibitor families (Figure 2) utilized the well-described compound targets to create a non-biased filter in selecting which compounds to further peruse. With only 400 compounds, the OICR library is relatively small, prohibiting the use of rigorous statistical analysis. However, as a proof-of-concept, we demonstrate that even descriptive statistics can enhance the otherwise arbitrary selection of compounds of interest for downstream validation. Upscaling our screen methodology has the potential to provide invaluable data on which target families are good candidates for host-targeting compounds for *M.tb* infection, and can also be generalized to other infection models. Based on our observation, we hypothesize that focused screening using CHK, IGF-1R, and Akt inhibitors is likely to result in discovery of novel, highly active, and non-toxic HDTs. One signaling pathway that is both well-characterized in *M.tb* infections and enriched in our analysis is the PI3K-Akt-GSK3-mTOR pathway. Previous work from our lab (Poirier et al., 2014) and others (Singh and Subbian, 2018; Hu et al., 2020), as well as this work, highlight this pathway’s inhibition in controlling intracellular infection via control of cellular apoptosis and autophagy. Since signaling pathways commonly interreact with each other, upscaling our screen and analysis can highlight those sub-paths relevant to *M.tb* inhibition, providing a complimentary and unbiased approach to classical biochemical pathway characterization techniques.

DDUG and its target CHK2 were validated as inhibitory by a dose-dependency test and by using RNAi (Figure 3), respectively. Tandutinib, an inhibitor of three different kinases that was also validated, showed MIC_50_ value above 10 μM, making it an unlikely treatment candidate, and 10-DEBC was originally inspected as a novel compound and since reported by Han et al. (2019). For both DDUG and 10-DEBC, we were able to characterize their intracellular mode of inhibition as bactericidal and bacteriostatic, respectively, and demonstrated that while DDUG was not active against other pathogenic mycobacteria, 10-DEBC was non-specific. Of note, 10-DEBC is a member of the chlorpromazine family of compounds, which were demonstrated to inhibit *M.tb* (Raffel et al., 1960; Crowle et al., 1992) without being Akt inhibitors themselves. Rather than being an HDT, 10-DEBC seems to be concentrated by the macrophage, where it is suggested to bind calmodulin to disrupt calcium transport (Amaral et al., 2001).

The characterized role of CHK2 is the arrest of cell-cycle via CDC25A, activation of DNA damage-repair mechanisms, and promotion of apoptosis via p53 in response to double-stranded DNA damage (Cai et al., 2009). Knockdown of CDC25A was also previously reported to decrease intracellular *M.tb* burden in THP-1 cells (Jayaswal et al., 2010). Some human viruses, such as human T-cell leukemia virus 1 (Durkin et al., 2008) and Epstein-Barr virus (Choudhuri et al., 2007), were shown to modify CHK2 for their replicative needs. However currently, other than CDC25A there is little evidence tying these pathways with inhibition of *M.tb* growth intracellularly. The activity of DDUG against *M.tb* in broth at concentrations similar to those achieved intracellularly suggests an alternative, CHK2-independent mode of activity. Similarly, the activity of DDUG against some bacteria further suggest DDUG might have promiscuous activity on bacterial kinases, similar to NU-6027 (Kidwai et al., 2019) and AZD7762 (Kanehiro et al., 2018), or otherwise be toxic due to physical properties such as crystallization if concentrated by the macrophage. Conversely, DDUG is not active against *M.tb*’s close family members, *M. abscessus* and *M. bovis*-BCG, or *Salmonella*, which it did inhibit in macrophages, and is active against a small array of seemingly unrelated bacteria (Table 3). Since it is unlikely the compound specifically binds a molecular target shared between a subset of the bacteria tested but not the others, these observations do not support a bacterial kinase target mode of action hypothesis.

We are unable to decisively determine if DDUG is acting via a CHK2-dependant or independent mechanism. Supporting the first are independent inhibition of CHK2 by RNAi, inhibition by other library-screened CHK2 inhibitors (provided validation), specificity of DDUG to *M.tb* vs. *M. bovis*-BCG and *M. abscessus* in THP-1 cells, and the lack of CHK2 activity in BMDM. Supporting the latter are activity against *M.tb* (and 25% of other bacteria tested) in broth at concentrations only twofold higher than observed intracellularly. Of note, a literature-grounded hypothesis can support CHK2-dependant inhibition of *M.tb* intracellular growth. Examining an array of CHK2 inhibitors and further investigating the CHK2-CDC25A pathway can help resolve this challenge, as well as further examination of DDUG’s toxicity in *M. catarrhalis* and *S. epidermidis*, yet are beyond the scope of this screening campaign. However, this temporary lack of a definitive mode of inhibition does not take away from DDUG’s remarkable intracellular inhibition of *M.tb* with low associated cell loss, and the demonstrated effect CHK2 inhibition has on intracellular *M.tb* growth in macrophages.

Host-targeting compounds were also equally potent against the BCL2-safe auxotroph MC^2^ 6206 strain. This is particularly promising, as current screens against mycobacterial models (such as *M. bovis*-BCG, *Mycobacterium marinum*, and others) face complications struggles coming up with viable antibiotics for *M.tb*, complications that intensify with HDTs as *Mycobacterium* spp. adopt unique host-pathogen interactions. In demonstrating that the auxotrophic *M.tb* strain is susceptible to current HDTs, the prohibitive barrier of access to and automatization in BCL3 settings can be bypassed, with the auxotroph used for future screening campaigns and in studying compound modes of action. Clearly, findings from any auxotroph study will require corroboration in WT *M.tb* to account for the differences in metabolic activity between the strains, which may certainly have an effect on compound activity.

The exploration of HDT compounds against intracellular *M.tb* is gaining momentum, with this study and others offering promising findings on the ability to treat *M.tb* by targeting the host cell. However, as HDT can have synergistic, additive, or even antagonistic actions with current TB therapeutics, and for clinical relevancy, HDT should be tested in concert with current approved TB drugs.

## Conclusion

In conclusion, we demonstrated here how intelligible small scale, top-down screens can replicate and improve screening efforts of much larger, more expensive strategies. The key factors contributing to the success of the screen were its anchors in ground-level microbiology: (1) screening a reliable and valid intracellular niche model, (2) testing compounds that are well-characterized or in advanced clinical stages to expedite potential knowledge translation to meet clinical needs, and (3) investigating kinase signaling—processes quintessential to *M.tb* host-pathogen interactions.

## Data Availability Statement

All datasets presented in this study are included in the article/Supplementary Material.

## Author Contributions

TS designed, conducted, managed, and wrote the research. LR-W tested broth activity of DDUG in BCL3 and assisted with design and writing. JC performed RNAi validation and assisted with writing. VP tested broth activity of DDUG in BCL2. CR tested DDUG intracellular activity against *S. typhi* and assisted with design and writing. TP contributed with experimental design and analysis. YA-G proposed, designed, and initiated the study, obtained funding, and participated in writing. All authors contributed to the article and approved the submitted version.

## Conflict of Interest

The authors declare that the research was conducted in the absence of any commercial or financial relationships that could be construed as a potential conflict of interest.
