# Supplementary material for: High-Content Screening of Eukaryotic Kinase Inhibitors Identify CHK2 Inhibitor Activity Against Mycobacterium tuberculosis
Source: Front Microbiol. 2020 Sep 18;11:553962. doi: 10.3389/fmicb.2020.553962 (PMC7530171; doi:10.3389/fmicb.2020.553962)
Supplement: Supplementary file 3 [file Data_Sheet_1.PDF]

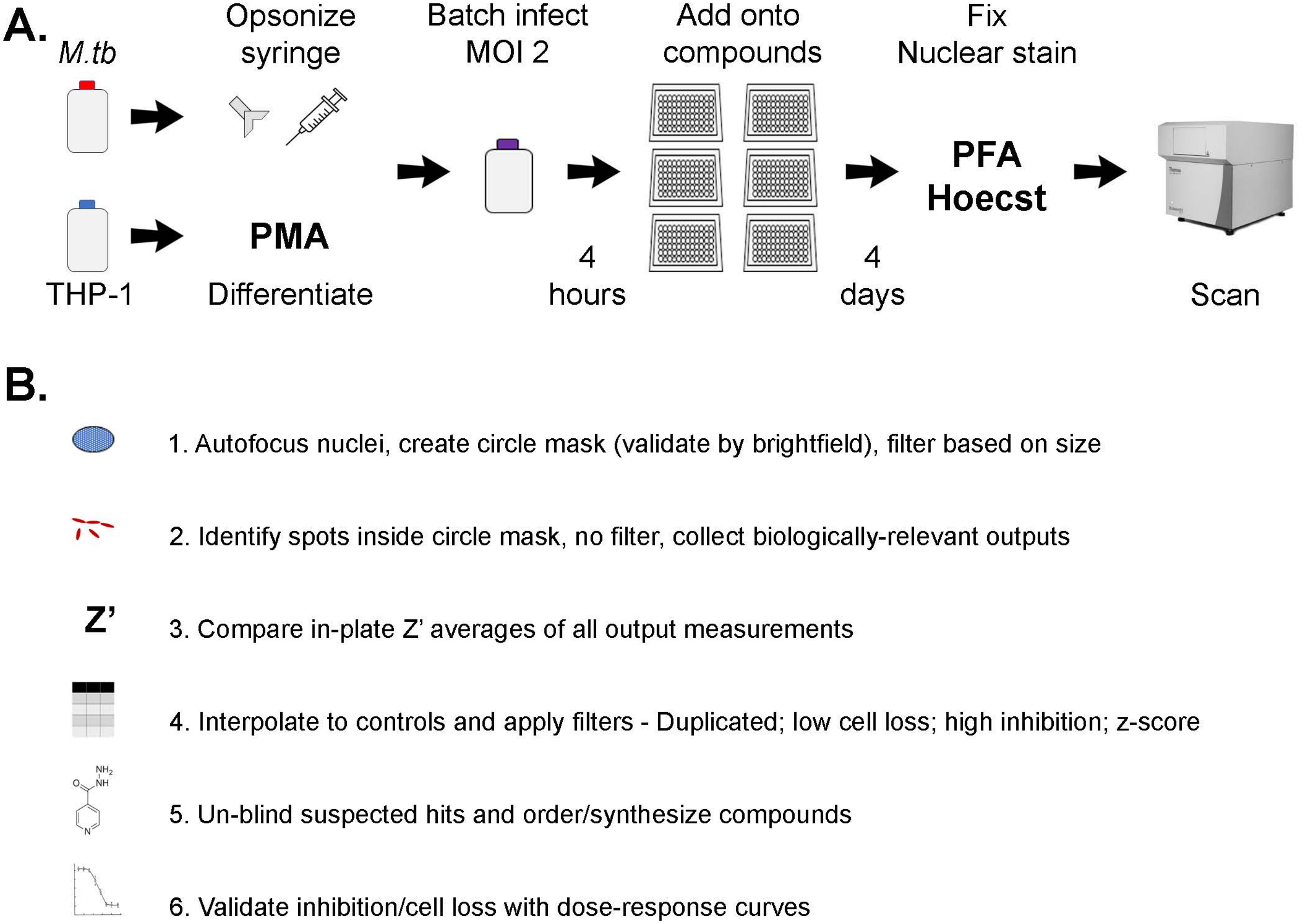

Supplemental Figure 1. Screen experimental design
M.tb-pTEC27 were used to infect PMA-differentiating THP-1 cells after bacteria were opsonized with human serum, and clumps disrupted using a 25G syringe. OICR library infected blinded, in independent duplicates. MOI, clump disruption, compound-treatment length, and nuclear stain were calibrated in preliminary studies to optimize readout (A). Analysis of scanned library steps (B). Size filter for step 1 excludeds cell clumps (>5 cells) and cellular debris (<20% average nuclei size). Step 2 outputs CircSpot Total/Average Area/Intensity, CircSpotCount, CircTotalIntensity. Step 3 Z’ averages ranged from -0.2 (CircSpotCount) to 0.5 (CircSpotTotalIntensity). The latter ranged between plates from 0.3 to 0.6. Step 4 filters decided on and applied before unblinding.

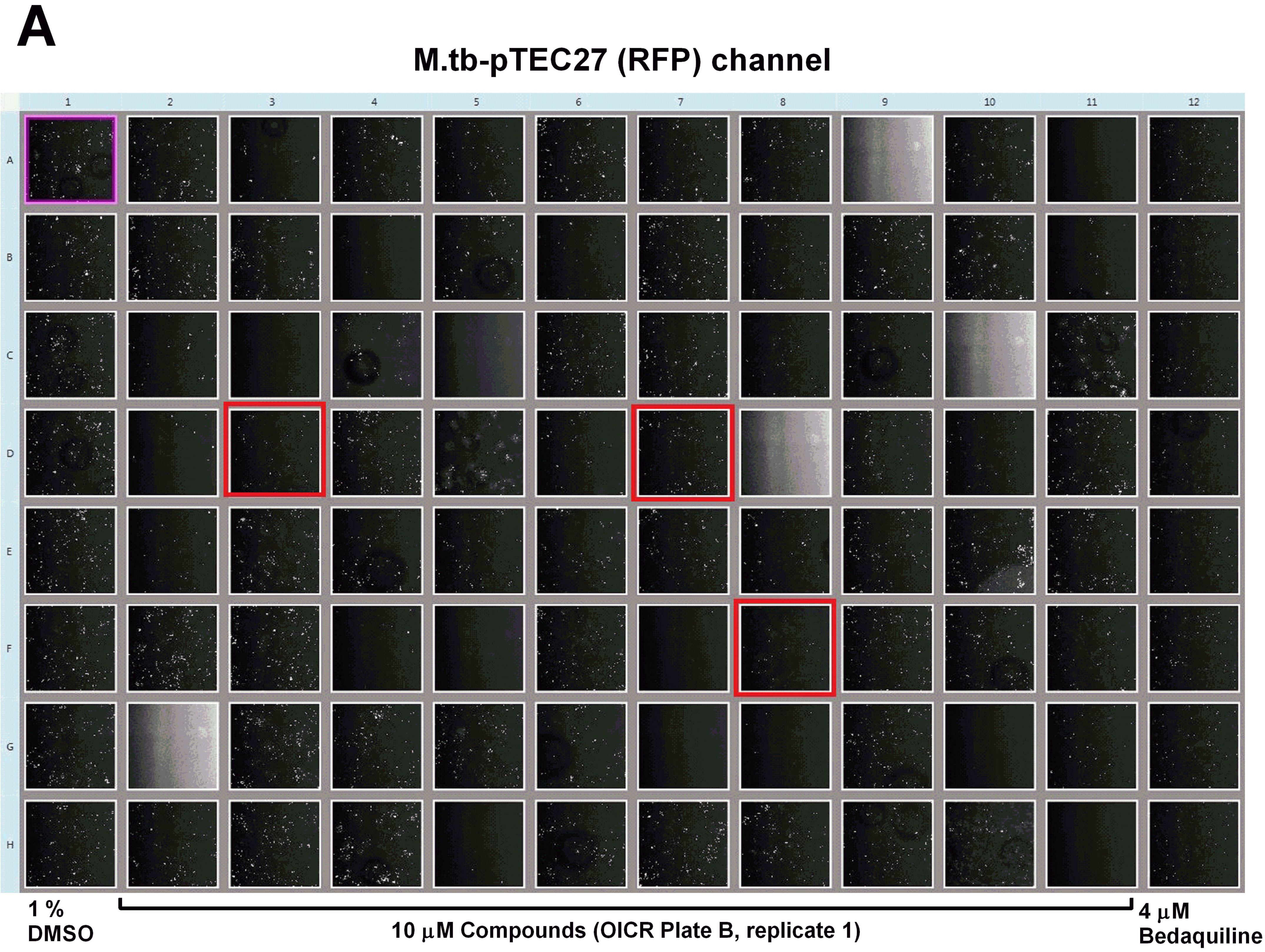
A
M.tb-pTEC27 (RFP) channel
1 2 3 4 5 6 7 8 9 10 11 12
A B C D E F G H
1 %
DMSO
10 μM Compounds (OICR Plate B, replicate 1)
4 μM
Bedaquiline

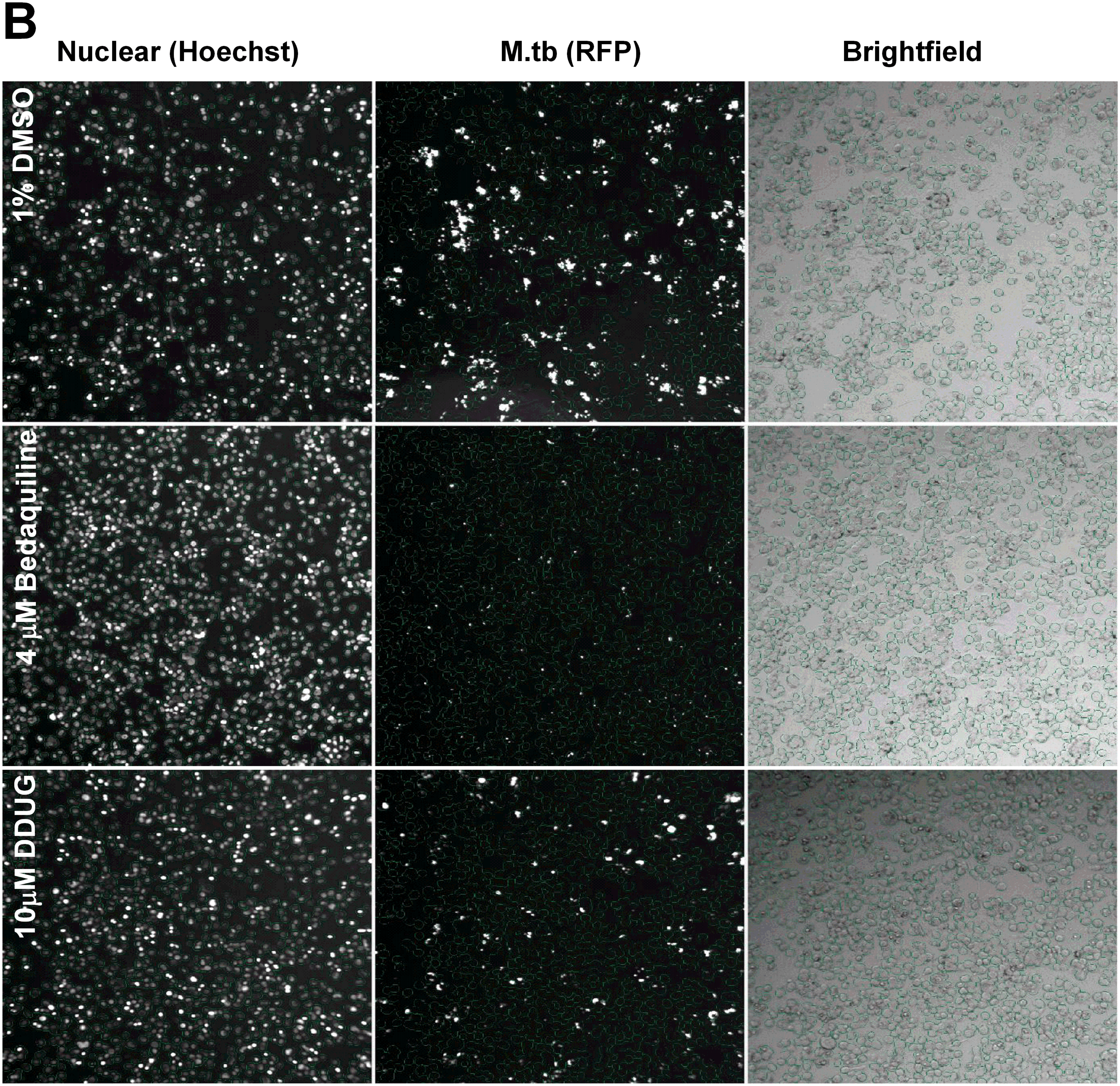
B
Nuclear (Hoechst)
M.tb (RFP)
Brightfield
1% DMSO
4 μM Bedaquiline
10μM DDUG

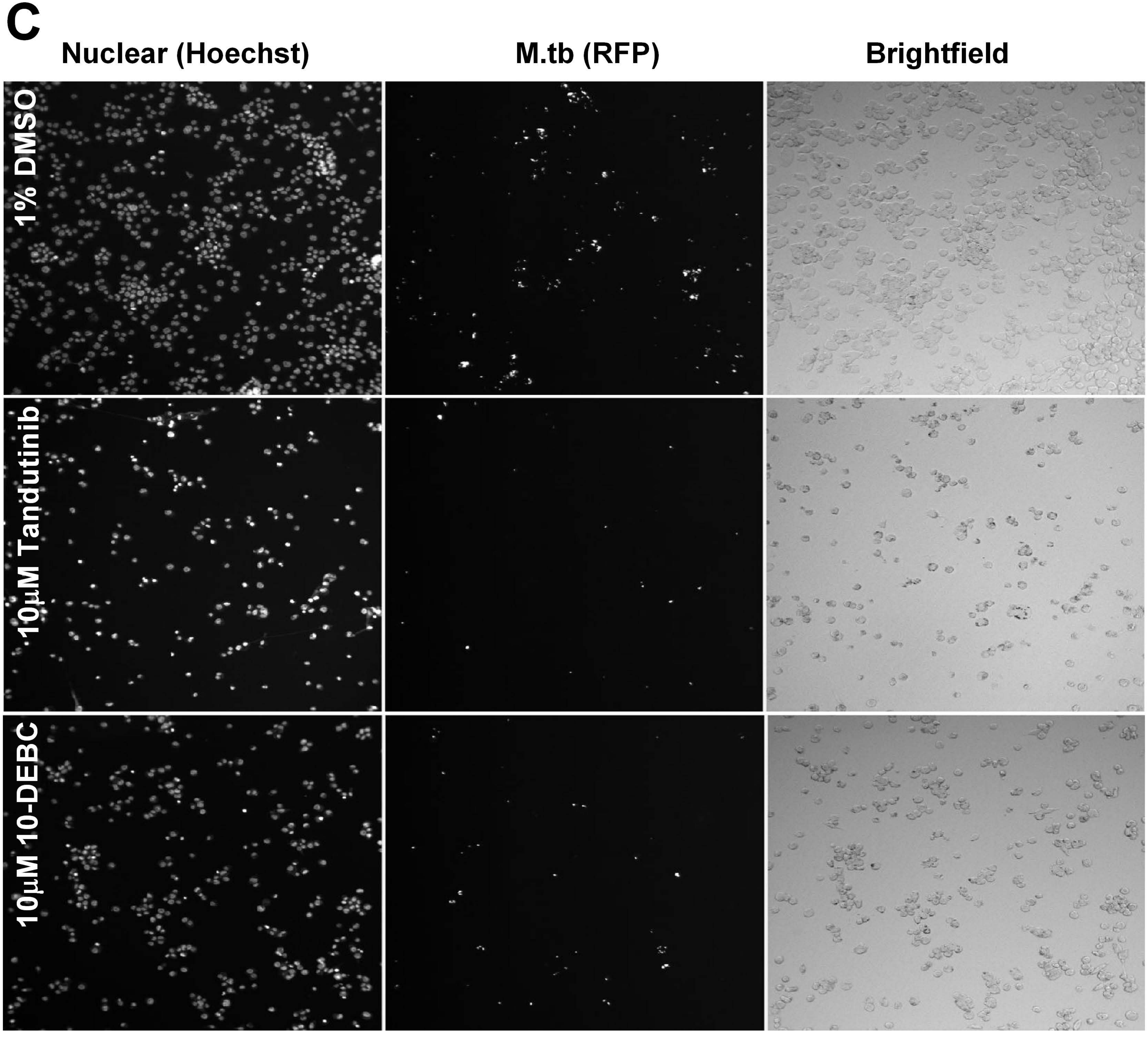
C
Nuclear (Hoechst)
M.tb (RFP)
Brightfield
1% DMSO
10μM Tandutinib
10μM 10-DEBC

Supplemental Figure 2. Representative High-Content scan images

RFP channel (*M*.tb-pTEC27) of one replicate of plate B (out of A-E), central field of 9 scanned per well (A). DMSO control (purple) in column 1 and Bedaquiline control in column 12. Bright readouts such as seen in well G2, D8, A9, and C10 occur when autofocus on nuclei fails due to complete cell loss. Wells such as D3, D7, and F8 (red) demonstrate low infection loads compared to controls. Mask creation (B) is based on nuclear-stained objects. A circle mask (green) is created to estimate cellular area, verified using brightfield image. RFP signal is quantified from mask areas only, excluding extracellular signal, or that coming from nuclei filtered for being to small or too large. Cell loss due to compound cytotoxicity or effect on cell adherence (C) quantified using nuclear-stained object numbers, normalized to the DMSO control. Images in B & C are one representative field from 9 scanned per well.
